# A Mixed‐Methods Investigation of the Role of Sense of Community on Older Adults’ Memory Self‐Efficacy and Control Beliefs Following an Online Group Memory Intervention

**DOI:** 10.1155/jare/1022137

**Published:** 2026-07-23

**Authors:** Duy P. Dao, Carl I. Moller, Christina Bryant, Kathryn A. Ellis, Kerryn E. Pike

**Affiliations:** ^1^ School of Psychology and Public Health, La Trobe University, Melbourne, Victoria, Australia, latrobe.edu.au; ^2^ Deakin Institute for Mental and Physical Health and Clinical Translation (IMPACT), School of Medicine, Deakin University, Melbourne, Victoria, Australia, deakin.edu.au; ^3^ Melbourne School of Psychological Sciences, The University of Melbourne, Melbourne, Victoria, Australia, unimelb.edu.au; ^4^ Department of Psychiatry, The University of Melbourne, Melbourne, Victoria, Australia, unimelb.edu.au; ^5^ John Richards Centre for Rural Ageing Research, La Trobe University, Wodonga, Victoria, Australia, latrobe.edu.au; ^6^ Griffith Centre for Mental Health & School of Applied Psychology, Griffith University, Gold Coast, Queensland, Australia, health.qld.gov.au

**Keywords:** control beliefs, memory self-efficacy, older adults, online memory intervention, sense of community

## Abstract

Using a mixed‐methods design, we investigated the effect of an asynchronous online group intervention for older adults with subjective cognitive difficulties on their memory self‐efficacy and control beliefs, in a pre–post single group study. We hypothesized that engaging with online discussion boards would foster a sense of community amongst participants, helping improve self‐reported memory self‐efficacy and control beliefs. One hundred and twenty‐seven community‐dwelling adults aged 60–88 years, drawn from the Online Personalized Training in Memory Strategies for Everyday (OPTIMiSE) study, completed questionnaires assessing memory self‐efficacy, control beliefs, sense of community and usefulness of the discussion boards. The number of discussion board posts was recorded to indicate engagement. Findings revealed significant improvements to memory self‐efficacy and control beliefs postintervention. Whilst not all participants reported a strong sense of community, their opinions of the discussion boards were generally favourable, particularly in terms of normalization of memory concerns through sharing with others. Time constraints, limited quality of interactions and decreased engagement were reported to impact both the usefulness of the discussion boards and the development of a sense of community. There are promising implications for the utility of online memory interventions in promoting greater confidence and feelings of control regarding memory ability through psychoeducation, strategy training and in providing reassurance through normalization.

**Trial Registration:** Australian New Zealand Clinical Trials Registry: ACTRN12620000979954

## 1. Introduction

Subjective cognitive concerns—particularly those focused on memory—are common in older adults [1] and are associated with feelings of distress [2] and increased risk of developing mild cognitive impairment and dementia [3, 4]. Various interventions have been developed to mitigate memory and other cognitive concerns [5–7]. This is commonly achieved through a combination of psychoeducation and either a restorative (to regain cognitive function) or compensatory approach (to optimize cognitive function through use of strategies; [8]). To date, memory interventions for older adults have generally been conducted face to face. There are, however, numerous access barriers associated with the provision of face‐to‐face services for older adults, such as mobility and transport issues, rural residence and disruptions to in‐person services during public health emergencies such as the COVID‐19 pandemic [9, 10]. A potential solution is to offer memory interventions online, and hence we recently developed and evaluated the OPTIMiSE [11, 12] programme. OPTIMiSE is a six‐module online memory course for older adults with subjective cognitive concerns, adopting a compensatory approach and utilizing psychoeducation to challenge negative perceptions about memory function in older age. OPTIMiSE demonstrated good feasibility (strong interest and fair attrition rate), acceptability (97% of participants who completed the programme would recommend OPTIMiSE) and efficacy (improvement for all primary outcome measures [memory goal satisfaction, strategy knowledge and use, self‐reported memory, memory satisfaction and knowledge and mood]; [11]), indicating this intervention could be an option to increase access to evidence‐based memory interventions to support older adults living with cognitive concerns.

Whilst online memory interventions like OPTIMiSE (and others, e.g., [9]) are feasible, acceptable and effective in changing views about memory in daily life, we do not yet know whether they impact memory self‐efficacy or memory control beliefs. Subjective memory concerns by older adults are commonly accompanied by reductions in *memory self-efficacy* (the level of confidence in one’s memory ability [13]) and weaker *control beliefs* (the degree of influence an individual believes they have over their memory [14]). Older adults are more likely to endorse negative evaluations about their memory competence [15] and hold views that memory decline is inevitable and resistant to change [16]. This can lead to avoidance of situations that depend on memory function [17], self‐doubt, reduced strategy use and concerns about failure [18–20], affecting memory performance on testing [21, 22] and increasing instances of apparent memory failures in everyday life [23]. These failures then reinforce beliefs that memory decline is uncontrollable [19], further diminishing self‐confidence in memory ability [24]. Adults who attribute memory outcomes to controllable factors have been found to outperform those who associate memory performance with age or natural ability, as they may engage in more effective and regular strategy use [25]. Interventions that target beliefs about memory may therefore be effective in improving self‐efficacy and control beliefs for memory in older adults [26], subsequently aiding memory performance and functioning [22, 27] and promoting better quality of life [28].

Previous interventions have shown that such beliefs are amenable to change, with the promotion of stronger control beliefs demonstrated through memory strategy training, psychoeducation and cognitive restructuring [29, 30]. To increase an individual’s confidence, it has been proposed that four factors contribute to self‐efficacy: mastery experiences (experiencing success), vicarious observations (seeing others succeed), social persuasion (receiving encouragement from others) and arousal states (one’s emotional responses to situations) [31]. The social aspects in face‐to‐face memory groups are thought to be important in improving self‐efficacy and control beliefs [32]. Past research has specifically focused on improving memory self‐efficacy using these four factors [31], finding that older adults who completed memory training in a group, rather than individually, reported improved self‐efficacy beliefs postintervention, with concurrent increases in recall performance and control beliefs [24]. Similar findings found that older adults who received group memory training demonstrated improved memory self‐efficacy, alongside increased stability and fewer feelings of stress and anxiety related to memory, compared to those who underwent individual memory training [17]. Furthermore, a meta‐analysis of 31 randomized controlled trials found that group interventions led to increased memory self‐efficacy ratings, better performances on half the memory measures assessed and reduced memory‐induced anxiety, compared to training in individual settings [33]. Group training also provides opportunities for normalizing memory concerns through shared experiences [2, 17]. For example, older adults completing the La Trobe and Caulfield Hospital (LaTCH) group‐based memory intervention [34] reported that initial feelings of worry about their memory changed into increased self‐confidence and a sense of control after programme completion [2]. When participants noticed that their memory difficulties were shared amongst others, this promoted feelings of social connectedness.

This concept can be referred to as *sense of community,* which relates to collective experiences and feelings of group belonging [35]. Strong social connections within a community are important and shown to be associated with enhanced well‐being [36] and social support [37]. Establishing and maintaining a sense of community is proposed to relate to four dimensions: *membership*—feelings of belonging to a group; *influence*—the impact members have on a group and vice versa; *shared emotional connection*—individuals can identify with the ‘history’ of other members; and *fulfillment of needs*—it must be rewarding to be part of the group [38]. Sense of community is thought to be linked with outcomes from group (rather than individual) participation.

Previous studies of online memory interventions have found that older adults report increased confidence in their memory after training [9, 11]. However, these studies only asked a single question after the training to assess participants’ confidence levels. A more in‐depth examination of change in self‐efficacy following the completion of an online memory intervention compared experimental and wait‐list groups [39]. It was reported that there were no differences between participants who had received the intervention compared to those who had not; however, the measure used was the General Self‐Efficacy Scale, which focuses on coping and problem‐solving generally in daily life, not memory specifically. To our knowledge, memory control beliefs have not been reported following an online memory intervention.

Early data are emerging regarding the effectiveness of online communities in promoting a sense of community, including those centred on experiences of disability [40], youth depression [41] and social anxiety [42]. Whether these findings generalize to older adults with reported cognitive complaints is yet to be investigated, although it has been reported that their participants would have valued the ability to socialize online [9].

The aim of this study was to determine whether an eight‐week asynchronous online group memory intervention (OPTIMiSE) for older adults with subjective memory concerns results in improvements in memory self‐efficacy and memory control beliefs, facilitated by a sense of community. It was hypothesized that self‐reported memory self‐efficacy and memory control beliefs would improve after completion of OPTIMiSE. Given the importance of social aspects in face‐to‐face interventions, online moderated discussion boards were implemented into OPTIMiSE to help promote a sense of community amongst participants, with better engagement predicted to be associated with higher ratings of sense of community. It was also hypothesized that participants would experience a sense of community, and that a stronger sense of community would be associated with improvements in memory self‐efficacy and memory control beliefs. The study also explored participants’ views on the usefulness of the discussion boards and creation of a sense of community, as these have not been investigated within an online memory intervention for older adults.

## 2. Method

### 2.1. Design

This was an eight‐week single‐arm study with outcome measures administered at baseline and postintervention. Although primarily a quantitative study, we included qualitative analysis of some open‐ended questions to explore the discussion board utility and creation of a sense of community. The study was reviewed and approved by the La Trobe University Human Research Ethics Committee (HEC20025) and the University of Tasmania Ethics Committee (HEC20323). The study was also registered with the Australian New Zealand Clinical Trials Registry (ANZCTR). STROBE guidelines [43] have been followed for the reporting of the study.

### 2.2. Participants

Participants were part of a larger study investigating the suitability, feasibility and effectiveness of OPTIMiSE [11] and consisted of older adults (aged 60 and over) with subjective cognitive concerns (over the last 10 years). Participants were recruited through emails between 11 September 2020 and 8 October 2020, targeting different community groups across Australia, including University of the Third Age (U3A), Probus clubs and individuals who had previously expressed interest in the face‐to‐face LaTCH memory intervention but were unable to attend. Informed consent was provided by all participants prior to completion of any measures. Details of the recruitment and informed consent process have been published previously [11]. Inclusion and exclusion criteria for OPTIMiSE are listed in Table 1.

**TABLE 1:** Inclusion and exclusion criteria for OPTIMiSE^∗^.

Inclusion	Exclusion
‐ Aged 60 and over—self‐reported subjective cognitive decline over the last 10 years ‐ Sufficiently fluent in reading and typing English to access, read and comprehend the course material and participate in text‐based online discussions ‐ Able to complete the course within the specific 8‐week period ‐ Able to complete the online evaluation questionnaires without assistance ‐ Resident of Australia	‐ Preexisting diagnosis of: ◦ Dementia ◦ Psychiatric disorder likely to impact cognition ◦ Neurological condition likely to impact cognition or study participation ‐ History of drug or alcohol dependency

^∗^Inclusion and exclusion criteria were determined based on participant self‐report.

### 2.3. Intervention

Participants completed the six‐module OPTIMiSE course over an eight‐week period, with each module consisting of approximately 2 hours of content. Participants were able to complete the modules at any time during this 8‐week period. An overview of the module content has been published previously [12]. In brief, modules covered models of memory, memory changes across the lifespan (i.e., memory in normal ageing vs. dementia), evidence‐based memory strategies and modifiable lifestyle changes to improve memory function.

### 2.4. Outcome Measures

Participants were required to complete relevant outcome measures assessing memory self‐efficacy and control beliefs prior to commencing OPTIMiSE. Follow‐up questionnaires could be immediately accessed by participants after completion of the programme. A maximum of 3 weeks was provided (depending on when participants finished all six OPTIMiSE modules) for participants to complete the postintervention measures.

#### 2.4.1. Memory Self‐Efficacy

Preexisting memory self‐efficacy measures were not used as they either had too many items, increasing participant burden (Memory Functioning Questionnaire [64 items]; [44]), irrelevant items not covered by OPTIMiSE training (Memory Self‐Efficacy Questionnaire [45]), or were too broad in scope (Metamemory in Adulthood Questionnaire [46]). These issues were addressed by designing a concise, novel eight‐item measure of memory self‐efficacy (see Supporting Information A) to assess the level of confidence around (1) overall memory functioning and (2) memory for specific day‐to‐day situations (e.g., remembering names of people). These memory situations were selected to match the strategies covered in OPTIMiSE, which largely overlap with items from other self‐efficacy measures such as the Memory Functioning Questionnaire [44] and Memory Self‐Efficacy Questionnaire [45]. Responses were recorded on a Likert‐type scale ranging from 0 = *not confident at all* to 10 = *very confident*. Cronbach’s alphas were calculated for the seven items relating to specific memory scenarios using data from preintervention (*α* = 0.86, *M* = 6.14, *SD* = 0.82) and postintervention (*α* = 0.86, *M* = 7.03, *SD* = 0.61) and were deemed acceptable according to conventional criteria [47]. The average of these seven items was used as an overall measure of memory self‐efficacy related to OPTIMiSE. Exploratory factor analysis (EFA) using Oblimin rotation was conducted on the baseline data for the seven items assessing memory for specific day‐to‐day situations. Using the conventional eigenvalue cutoff of > 1, the EFA suggested a 1‐factor solution, with all seven items highly loading onto a single factor (all factor loadings were > 0.60). This suggested that the observed data were better accounted for by a single underlying latent construct. Mean interitem correlation across the seven items was 0.48.

#### 2.4.2. Control Beliefs

To assess control beliefs, the *Memory Controllability Inventory* (*MemCo* [27]) was administered at baseline and postintervention. The *MemCo* consists of 19 items and was designed to measure beliefs about memory ability and the controllability of memory. There are six subscales: *Present Ability* (beliefs about current memory ability)*, Potential Improvement* (degree of confidence in the effectiveness of strategies on memory)*, Effort Utility* (how much one attributes effort to memory function)*, Independence* (one’s ability to manage their memory without assistance from others), *Inevitable Decrement* (the degree to which one believes memory inevitably declines with age) and *Alzheimer’s Disease Likelihood* (belief that developing Alzheimer’s disease is inevitable with age) and responses range from 1 (*strongly disagree*) to 7 (*strongly agree*). Responses to items within each subscale were averaged to generate a score. Items from the *Inevitable Decrement* and *Alzheimer’s Disease Likelihood* subscales were reverse‐scored, so that higher scores on all six subscales would reflect stronger control beliefs for memory ability. All subscales were then averaged to create an overall MemCo score. Previously reported Cronbach’s alphas for the individual subscales ranged from 0.49 to 0.77 [27].

#### 2.4.3. Sense of Community

Two measures were used to assess sense of community following the completion of OPTIMiSE, a modified version of *The Brief Sense of Community Scale* (*BSCS* [35], where the term ‘neighbourhood’ in each item was replaced with ‘online community’) and a follow‐up evaluation form (see Supporting Information B). The *BSCS* contains eight items covering the four dimensions proposed to relate to sense of community [38]: *Membership*, *Influence*, *Fulfillment of Needs* and *Emotional Connection*, rated on a 5‐point Likert scale ranging from 1 (*strongly disagree*) to 5 (*strongly agree*). An average rating greater than 3 (with 3 indicating that a participant was unsure/neutral) was required to demonstrate that a sense of community was established. The overall *BSCS* has previously been shown to have adequate reliability (Cronbach’s alpha was 0.92 (*M* = 3.81, *SD* = 0.79; [35]). The wording of the items was adapted for the online community in this study, as the items from the original scale referenced physical neighbourhoods. The follow‐up evaluation form (Supporting Information B) included seven questions pertaining to participants’ experiences with sense of community and using the online discussion boards. The form required a combination of written responses and ratings on 5‐point Likert scales (1 = *strongly disagree* to 5 = *strongly agree*).

### 2.5. Discussion Board Engagement

After each module, participants were asked to reflect on the information presented and report on the strategies they had practiced on the moderated online discussion boards (there were 38 discussion boards in total). Access to specific discussion boards was only available to participants following successful completion of the relevant core module or optional content. All participants contributed to the same discussion within each discussion board. The discussion boards were asynchronous in nature, meaning posts and responses to posts did not occur in real time (posts could be made at any time by participants until a discussion board was closed). Level of engagement on the discussion boards was measured by summing the number of posts and replies participants made across all discussion boards. There was no limit on how many posts could be made.

### 2.6. Statistical Analysis

Statistical analyses were conducted using Jeffrey’s Amazing Statistics Program (JASP; Version 0.14.1.0). To examine changes in memory self‐efficacy and control beliefs between pre and postintervention, paired samples *t*‐tests were conducted. To determine if changes to memory self‐efficacy and control beliefs were associated with sense of community, the difference in ratings between baseline and postintervention for memory self‐efficacy (average of seven specific items) and control beliefs were correlated with sense of community ratings (on the *BSCS*). Pearson correlations were used to determine whether there was a relationship between the level of engagement (total number of posts and replies made on the discussion boards) and reported sense of community levels. Cohen’s *d* was used to measure effect sizes for all correlations, which were interpreted in accordance with conventional guidelines (small = 0.2; medium = 0.5; large = 0.8; [48]). The Bonferroni correction method [49] was applied to adjust for potential inflated Type I error from multiple comparisons.

### 2.7. Qualitative Analysis

Whilst this study was primarily focused on quantitative measures, the addition of qualitative data added further depth to the findings, especially for discussion board use and sense of community outcomes, which have not been thoroughly investigated in the context of a memory‐based intervention. Participants were asked to complete a follow‐up evaluation form (see Supporting Information B) about their experiences of sense of community and using the online discussion boards. Questions that required participants to provide additional written responses included ‘Why did/didn’t you find the discussion boards useful?’ and ‘Why do you feel that a sense of community was/was not created amongst others with similar memory concerns?’ It was not compulsory for participants to respond to these questions. Author D.D (Clinical Neuropsychology master’s student) reviewed all responses using an interpretivist approach and coded these into common themes [50]. Subsequent consultation with author K.P (Clinical Neuropsychologist and researcher) was conducted, where a consensus about the themes was reached.

## 3. Results

### 3.1. Participants

From an original pool of 387 participants who provided informed consent to participate in OPTIMiSE, 357 participants passed eligibility screening and completed all baseline questionnaires. In this substudy, only participants who completed relevant measures of interest at both baseline and postintervention were included (*n* = 127). Participant demographics are outlined in Table 2, with no significant differences on any measures between the ‘Completers (completed study measures at baseline [prior to intervention] and postintervention time points)’ and ‘Baseline Only (completed study measures at baseline only)’ groups.

**TABLE 2:** Comparison of demographics for completers and baseline only samples.

	Completers (*n* = 127)	Baseline only (*n* = 230)	Comparison by completion status	*p* value
Age in years (*m* (sd))	72 (6.10)	72 (6.74)	*t* (126) = 0.94	0.35
Gender (female %)^1^	81.10	76.96	*X* ^2^ = 1.26	0.53
Education (greater than year 12 qualification %)	91.34	85.22	*X* ^2^ = 2.78	0.095
Experience with computers and browsing the Internet (‘moderate experience’ and above %)^∗^	91.34	87.83	*X* ^2^ = 1.04	0.31
Experience with online learning and courses (‘moderate experience’ and above %)^∗^	38.58	35.65	*X* ^2^ = 0.30	0.58
Family history of dementia or memory problems (yes %)	45.67	42.17	*X* ^2^ = 0.41	0.52
General health compared to others their age (‘average’ and above %)^∗∗^	96.06	95.65	*X* ^2^ = 0.03	0.85
General memory compared to others their age (‘average’ and above %)^∗∗^	84.04	86.52	*X* ^2^ = 0.15	0.70

*Note:* All *p* values in this set of comparisons exceeded the Bonferroni‐adjusted critical value (*α* level of 0.00625), and there was no evidence of any statistical difference across these demographics across the two groups. Completers = Participants who completed relevant measures at both pre and postintervention. Baseline Only = Participants who only completed relevant measures preintervention.

^1^One participant from ‘Baseline Only’ responded ‘prefer not to answer’ to the question of gender.

^∗^Determined based on Likert‐type scale with responses ranging from 0 (*no experience*) to 3 (*a lot of experience*).

^∗∗^Determined based on Likert‐type scale with responses ranging from 0 (*poor*) to 4 (*excellent*).

### 3.2. Changes in Memory Self‐Efficacy and Control Beliefs Following OPTIMiSE

At baseline, average levels of confidence for overall memory functioning and memory in specific everyday situations were slightly above the moderately confident range on the eight‐item measure of memory self‐efficacy. These ratings improved postintervention for both outcomes, with identical Cohen’s *d* effect sizes, which were of medium strength (see Table 3).

**TABLE 3:** Memory self‐efficacy pre‐ and post‐OPTIMiSE.

	Baseline *M* (SD) (*n* = 127)	Postintervention *M* (SD) (*n* = 127)	Comparison of pre‐ vs postratings	*p* value	Cohen’s *d*
Overall memory functioning	6.28 (1.69)	7.28 (1.37)	*t* (126) = 8.46	< 0.001	0.75 [0.55 to 0.95]
Memory in specific everyday situations	6.14 (1.57)	7.03 (1.35)	*t* (126) = 8.47	< 0.001	0.75 [0.55 to 0.95]

*Note:* Higher scores indicate greater memory self‐efficacy. 0 = not confident at all; 5 = moderately confident; 10 = very confident.

Regarding control beliefs, to account for multiple comparisons, a Bonferroni correction method was employed, with an adjusted *α* level of 0.0083. The results were statistically significant after correction, with improvements in ratings postintervention across all *MemCo subscales* except for *Effort Utility*. Means, standard deviations and effect sizes for each subscale are presented in Table 4. All statistically significant subscales demonstrated a medium to large effect (Cohen’s d ranging from 0.66 to 0.84). There was also a significant improvement seen for the overall *MemCo* score.

**TABLE 4:** Control beliefs pre‐ and post‐OPTIMiSE.

	Baseline *M* (SD) (*n* = 127)	Postintervention *M* (SD) (*n* = 127)	Comparison of pre‐ vs postratings	*p* value	Cohen’s *d*
Present Ability	4.66 (1.18)	5.34 (1.03)	*t*(126) = 7.85	< 0.001	0.70 [0.50 to 0.89]
Potential Improvement	5.24 (0.90)	6.07 (0.72)	*t*(126) = 9.45	< 0.001	0.84 [0.64 to 1.04]
Effort Utility	5.42 (0.75)	5.58 (0.92)	*t*(126) = 2.04	0.043	0.18 [0.01 to 0.36]
Inevitable Decrement	4.72 (1.10)	5.53 (1.00)	*t*(126) = 7.41	< 0.001	0.66 [0.47 to 0.85]
Independence	4.49 (1.11)	5.19 (1.04)	*t*(126) = 8.12	< 0.001	0.72 [0.52 to 0.91]
Alzheimer’s Disease Likelihood	4.58 (1.08)	5.49 (1.11)	*t*(126) = 9.09	< 0.001	0.81 [0.61 to 1.01]
Overall MemCo	4.85 (0.67)	5.53 (0.66)	*t*(126) = 12.29	< 0.001	1.09 [0.87 to 1.31]

*Note:* 1 = strongly disagree; 4 = neutral; 7 = strongly agree. Higher scores indicate stronger control beliefs.

### 3.3. Sense of Community in OPTIMiSE

Based on responses on the *BSCS*, at the completion of OPTIMiSE, 41% of participants endorsed experiencing a sense of community (i.e., average rating > 3), 50% did not experience a sense of community (rating < 3) and 9% were ‘unsure’ (rating = 3). On the follow‐up evaluation form, 52% of participants agreed that ‘a sense of community was created with others experiencing similar memory concerns’ (14% disagreed and 34% were unsure).

A thematic analysis was conducted for participants’ responses regarding their experiences with a sense of community and using the discussion boards. Overall, the same four themes were identified from the data on sense of community experiences and experience with the discussion boards. Three themes were identified describing reasons why participants felt a sense of community was not created: insufficient time, limited quality of interactions and reduced engagement (Table 5). These same themes were consistent across participants reported discussion board experience. A single theme emerged across both discussion board and sense of community experiences, where participants endorsed feelings of reassurance from the sharing and normalization of memory difficulties with other OPTIMiSE participants, also shown in Table 5.

**TABLE 5:** Sense of community and discussion board experiences (*n* = 127).

Themes	Examples related to sense of community experiences	Examples related to discussion board experiences
Insufficient time: Participants reported a lack of time or were too busy to read all the comments on the discussion boards.	‘Due to time constraints I was unable to read sufficient comments to get a sense of community’. ‘Lack of time prevented me from giving 100%’.	‘Too time consuming to work through’. ‘I found I didn’t really have time to get into the discussion boards’.

Quality of interactions: Participants reported that the quality of interactions was limited, particularly due to the asynchronous and delayed nature of the online discussion boards.	‘You simply can’t replace face‐to‐face interaction’. ‘In a face‐to‐face setting, participants would get instant feedback from others in the group’.	‘Would have preferred more interactive and personal discussions, e.g., small Zoom breakout groups’.

Limited engagement: Some participants were not motivated to consistently contribute to and utilize the discussion boards.	‘I never really felt part of the community and was a bit reluctant to share much’.	‘I often had nothing new to add to existing comments’.

Reassurance: Participants reported being reassured through the sharing and normalization of memory difficulties by others on the discussion boards.	‘I was greatly reassured to find these memory issues seem to be universal. And we’re all trying to improve and using similar strategies’.	‘Was beneficial to read that others had similar experiences with memory glitches’.

### 3.4. Association Between Sense of Community and Discussion Board Engagement

On average, participants made 17.46 posts (*SD* = 11.52) across all discussion boards throughout OPTIMiSE. The number of posts per participant ranged from 0 to 49, with 98% of participants posting at least once. In contrast to expectations, there was no significant correlation between the total number of discussion board posts and sense of community ratings (on the *BSCS*), *r* = 0.16, *p* = 0.075, 95% CI [‐0.02, 0.32].

On the follow‐up evaluation form, the majority of participants responded favourably towards most items assessing the potential benefits of using the discussion boards, with the exception of the item ‘Participants provided support, encouragement and positive feedback to one another’ (approximately 49% of participants agreed or strongly agreed; Table 6). Around 69% of participants tended to agree or strongly agree that the discussion boards were a useful resource, with feelings of reassurance through the sharing and normalization of memory concerns being a key explanation. The same three reasons for participants not experiencing a sense of community also emerged for the discussions being less useful (time, quality of interactions and engagement).

**TABLE 6:** Participant ratings of the online discussion boards (*n* = 127).

	Disagree/strongly disagree (%)	Unsure (%)	Agree/strongly agree (%)
The discussion board was a useful resource overall	5.51	25.20	69.29
It was helpful to get ideas from other participants on strategies I could try myself	7.87	27.56	64.57
I felt motivated to practice and apply strategies to everyday life after seeing them work for other participants	8.66	25.20	66.14
It was reassuring to read about other participants’ personal experiences relating to their memory difficulties and failures	2.36	13.39	84.25
Participants provided support, encouragement and positive feedback to one another	13.39	37.01	49.61
I felt less anxious about my memory abilities after engaging with the discussion board content	8.66	21.26	70.08

### 3.5. Association Between Change in Memory Self‐Efficacy and Control Beliefs With Sense of Community

There was a significant, yet small, positive correlation between the amount of improvement in memory self‐efficacy and sense of community (*BSCS* ratings). In contrast, no significant correlations were observed between any memory controllability inventory subscale and sense of community. Similarly, there was no significant association found when using the overall *MemCo* score. A summary of these results can be seen in Table 7.

**TABLE 7:** Correlations between change in memory self‐efficacy and control beliefs with sense of community.

	*r*	*p* value	95% CI
Memory Self‐efficacy	0.25	0.005	[0.08, 0.41]
Present Ability	0.06	0.50	[−0.12, 0.23]
Potential Improvement	0.005	0.95	[−0.17, 0.18]
Effort Utility	0.006	0.95	[−0.17, 0.18]
Inevitable Decrement	−0.04	0.66	[−0.21, 0.14]
Independence	0.12	0.19	[−0.06, 0.29]
Alzheimer’s Disease Likelihood	0.002	0.98	[−0.17, 0.18]
Overall MemCo	0.01	0.69	[−0.14, 0.21]

## 4. Discussion

This study aimed to investigate whether an asynchronous online memory intervention for older adults (OPTIMiSE) improved memory self‐efficacy and memory controllability, and whether this was related to the sense of community created via discussion boards. As predicted, participants reported increased memory self‐efficacy and stronger control beliefs following OPTIMiSE. Approximately half the participants reported experiencing a sense of community during OPTIMiSE. Sense of community ratings were not associated with the frequency with which participants posted on the discussion boards, nor did they correlate with the amount of improvement in control beliefs postintervention. In contrast, there was a small and significant correlation between sense of community ratings and improvement in memory self‐efficacy after completion of the programme.

### 4.1. Changes in Memory Self‐Efficacy and Control Beliefs Following OPTIMiSE

The results suggest participants became more confident and developed a greater sense of control over their memory abilities following OPTIMiSE. This was possibly attributable to both psychoeducation and memory strategy training provided. Modules were designed to help participants understand modifiable causes for their memory difficulties and highlight differences in memory changes between normal ageing and dementia. Participants were less likely to view memory decline as inevitable with age (*Inevitable Decrement*) and had reduced fears about developing Alzheimer’s Disease (*Alzheimer’s Disease Likelihood*) after completing these modules. This was consistent with previous research demonstrating that psychoeducation regarding age‐related memory decline leads to improved control beliefs, at least in an in‐person setting [51]. Participants in OPTIMiSE also reported increased confidence in using memory strategies (*Potential Improvement*) and felt less likely to rely on others for their memory (*Independence*). Memory training has been shown to enhance control beliefs by providing opportunities to learn and master strategies [29]. Participants experienced some success using strategies from OPTIMiSE (see [11]), which subsequently reinforced views that memory function relates to controllable factors. Since participants were encouraged to practice memory strategies (*social persuasion*), could complete modules at their own pace, set their own goals and expectations, and there was no emphasis on memory performance, this possibly reduced feelings of anxiety (*arousal states*). By integrating these elements proposed to increase memory self‐efficacy [31], experiences of memory failure and resulting anxiety, which negatively affect memory performance [21] were mitigated. This improvement in memory self‐efficacy is akin to that reported in previous face‐to‐face studies [17, 24]. Interestingly, participants did not endorse beliefs that effort was associated with memory improvement (*Effort Utility*). A possible explanation is that whilst OPTIMiSE participants can appreciate that their memory can improve, they may still perceive that a lot of effort and energy is still required to achieve this. Whether participants feel they are capable of sustaining this effort, particularly in their day‐to‐day life after the programme has ended, could be contributing to their evaluations.

### 4.2. Sense of Community in OPTIMiSE and Association With Online Discussion Boards

Overall, most participants rated the discussion boards favourably, yet only half the sample felt that the discussion boards were effective in facilitating a sense of community. Indeed, there was no significant association between engagement with discussion boards and sense of community ratings. For those who agreed that a sense of community was created through the discussion boards, a common theme relating to feelings of reassurance was evident. These participants felt that it was beneficial to recognize that their memory concerns were shared amongst other OPTIMiSE users.

There are several possible reasons why other participants did not endorse experiencing a sense of community. For example, it was reported that engagement and contribution to the discussion boards varied according to the available time to read through all the posted comments. In combination with the asynchronous nature of the discussion boards, participants also reported that the quality of interactions was reduced compared to those that would occur in a face‐to‐face setting. This is consistent with research indicating that older adults may have a preference for in‐person over Internet‐based interventions [52]. The extent to which a sense of community was elicited in OPTIMiSE might have been limited by not enough participants utilizing the discussion boards regularly to report a benefit, and limited opportunities for more synchronous communication with other users. Alternatively, it may be that participants had different expectations or interpretations regarding the nature of a sense of community and may have had difficulties applying this to an online setting. It is also plausible that the discussion boards were not sufficient to create a sense of community and that face‐to‐face interactions are necessary, at least in older adults with cognitive concerns, for there to be stronger social connections and leading to a stronger sense of community. Further, the level of engagement with the online discussion boards was only measured by the frequency of posts made by participants. A limitation of this approach is that it does not appreciate participants who may have engaged with the discussion boards more passively (e.g., by reading and reflecting on the content). Additionally, the depth or richness of engagement cannot be adequately captured by measuring the number of posts alone. It is acknowledged that other variables, such as the number of posts that were read, the amount of time spent on the discussion boards, and the quality or content of participants’ posts, are also plausible measures of engagement. Whilst this was beyond the scope of the current study, it does highlight interesting considerations for future research. Another possible explanation for the finding, however, is social desirability bias. Because the sense of community was assessed by self‐report, participants’ responses may have been influenced by a tendency to present themselves in a favourable or socially acceptable way. Socially desirable responding has been shown to obscure relationships between variables in questionnaire‐based research [53]. This possibility may be particularly worth considering in an older‐adult sample, given evidence that social desirability and community sense can be relevant to emotional outcomes in ageing populations [54].

Modifications to the online discussion boards might boost participant interaction and engagement, which may lead to a greater sense of community. For example, reducing the number of discussion boards (there were 38 in total in OPTIMiSE) would be more manageable for participants. To address issues relating to a lack of immediate feedback and long delays between posts, discussion boards could be only open at allocated times. This may result in a larger number of participants using the discussion boards at the same time and subsequently reading and responding to others’ posts in a timelier manner. In addition, participants should be encouraged to ask their own questions rather than just commenting in response to predetermined topics from OPTIMiSE. Questions posed by participants would open opportunities for others to contribute across a wider range of topics, more in line with their interests. Participants could also be asked to post on the discussion boards as a requirement to access the proceeding modules. Future research could also consider the demographic characteristics that may predict discussion board engagement (e.g., age, gender, education, experience with technology and online memory courses, and degree of concern about memory), particularly if the previously suggested modifications to the discussion boards are not successful in improving participant interaction online.

### 4.3. Association Between Memory Self‐Efficacy and Control Beliefs With Sense of Community

Partially supporting predictions, improvement in memory self‐efficacy, but not control beliefs, was associated with sense of community ratings. This may be explained by participants who reported a sense of community being more likely to acknowledge the benefits of shared experiences and subsequent normalization of their memory difficulties from OPTIMiSE. Whilst this does not confirm that sense of community adequately reflects the benefits of group involvement, it may suggest they are associated in some way. These collective experiences alongside psychoeducation help participants recognize that their memory difficulties are common and possibly challenge preexisting perceptions about memory decline in older age, subsequently promoting self‐efficacy [2, 12].

The findings that control beliefs were not associated with a sense of community align with the lack of research available in this area. Although studies have shown concurrent improvements to memory self‐efficacy and control beliefs [24], the impacts of group training and the associated social benefits are not as well established for control beliefs. Our findings suggest that the psychoeducational and strategy training aspects of OPTIMiSE, rather than the sense of community experiences, may have had a more significant role in strengthening control beliefs. The direction and nature of the association between control beliefs and memory self‐efficacy is unclear, as they do not always covary [55, 56], with discrepancies in the results potentially highlighting different underlying processes involved in driving the improvements participants are reporting for both concepts. That is, sense of community may not be related to how ‘in control’ people feel about their memory but rather influences how they generally feel about their memory function (more in line with self‐efficacy).

The correlation observed between sense of community and memory self‐efficacy, but not control beliefs, could also reflect the distinction between different types of memory‐related beliefs or reference frames [57, 58]. That is, older adults are reported to judge their memory function more favourably when comparing themselves to others rather than their own previous ability. This possibly highlights why sense of community experiences and opportunities to share and validate memory‐related challenges with other participants could influence more positive appraisals regarding the level of confidence in one’s memory ability (or self‐efficacy). In contrast, social comparisons potentially have less of an impact on altering control beliefs, which represent relatively more stable and long‐term perceptions about memory change in older age [55]. This is highlighted by the persistent age‐related differences reported in the literature, where older adults are more likely to hold views about reduced control over their memory [16]. It has been proposed that self‐efficacy can be optimised through contextual factors (mastery experiences, vicarious observations, social persuasion and arousal states [31]) and that this can be facilitated through social means [24], as supported by our findings.

## 5. Implications

The current study demonstrated that many participants found it beneficial to read about relatable experiences of memory difficulties from others. This suggests that normalizing these discussions in day‐to‐day life may help older adults feel more confident in their memory ability. Low levels of confidence and feelings of reduced sense of control may not only affect memory function but could also prevent older adults from reporting their difficulties out of fear of being negatively perceived or believing nothing can be done. This can be detrimental, particularly if their memory issues are associated with psychological distress or a reversible medical condition (e.g., thyroid issues; urinary tract infection) and intervention is delayed. Normalizing experiences and reducing anxiety around their memory may lead to older adults feeling more confident to seek further support for their memory, when required. If there truly are significant concerns about memory function, more accurate assessment and better diagnostic clarity may be achieved if the impact of anxiety and lowered confidence about memory can be mitigated. Importantly, this has been demonstrated through an online programme, highlighting the utility of online groups for increasing accessibility to memory interventions for older adults.

### 5.1. Strengths and Limitations

A strength of note was our large sample size, which consisted of older adults from across Australia. However, OPTIMiSE was mainly advertised to members of U3A, who tend to be highly motivated and well‐educated, with many possessing university‐level qualifications. It was therefore expected that our cohort would be more familiar with self‐directed learning and thus may have more experience with practicing novel strategies and completing modules independently compared to a less educated sample [59]. Indeed, people with less education may be expected to begin with lower memory self‐efficacy and may therefore potentially benefit to a greater extent. Additionally, all participants had experience using computers and browsing the Internet and the majority reported previous exposure to online learning and courses. Together, these variables highlight that the current sample is likely not fully representative of all older adults in the community. For future studies, avenues of recruitment should be broadened to include hospitals, memory clinics and general practitioners, as they serve people from more diverse backgrounds, with a wider variety of educational attainment. Another potential limitation relating to our sample was that there was a large proportion of participants who did not complete the entire OPTMiSE programme (i.e., the ‘baseline only’ group) and were therefore not included in our final analyses. The main reasons for participant withdrawal have previously been identified in the larger OPTIMiSE study [11] and included: participants experiencing technological difficulties, the high time commitment required and the content not being applicable to participants. For the latter, participants may have decided to withdraw from OPTIMiSE because they did not consider the programme to be beneficial, potentially resulting in a more positive evaluation of the programme overall from the remaining participants (i.e., the ‘completers’ group). Future studies should consider the following to help mitigate this issue (1) implementation of a systematic approach to adequately capture the reasons for participant withdrawal to inform modifications to the programme and minimize attrition and (2) incorporating an exit strategy for participants who do not complete OPTIMiSE, such as administration of briefer outcome measures that can still be used for postintervention analyses.

To assess memory self‐efficacy, a novel measure was administered to avoid constraints identified with preexisting measures. Whilst Cronbach’s alpha values were adequate, other psychometric properties, such as construct validity, were not investigated and could form the basis of future research. Results and aspects of the design were consistent with previous research targeting self‐efficacy in older adults [24, 60], providing some support that memory self‐efficacy was being appropriately measured.

Lastly, given the uncertainty around whether a sense of community accurately captures the social aspects of group training and the associated benefits, future studies could consider a more comprehensive qualitative approach. Whilst the present study was able to collect both quantitative and some limited qualitative data relating to participants’ experiences of sense of community, the latter only lent itself to a basic content analysis, as the way these questions were asked did not allow for free‐flowing responses that enable a deeper thematic analysis, which could help uncover relevant themes that better reflect the socially related benefits of group cognitive training.

## 6. Conclusion

An online memory intervention (OPTIMiSE) was found to increase older adults’ memory self‐efficacy and control beliefs, promoting more positive evaluations about their memory functioning. Whilst there was some evidence of normalization of memory difficulties through shared experiences from the online discussion boards, a sense of community was not established for approximately half the participants. Finding better ways to promote regular engagement on the discussion boards, alternative ways to facilitate sense of community experiences, and more appropriate measures to detect the social benefits of group training may add clarity to these findings. It is important that future revisions of OPTIMiSE consider a combination of advice from both experts in the field of memory research and intervention and previous OPTIMiSE users to determine which aspects of the programme will yield the most meaningful outcomes. OPTIMiSE provides some promising evidence to support more widespread use of online memory interventions, helping older adults to feel more confident and in control of their memory. Future revisions should explore whether these gains are maintained over time and lead to improved objective memory performance. It is hoped OPTIMiSE can be extended to more diverse populations to determine if other groups would similarly benefit.

## Funding

This work was supported by a La Trobe University Building Healthy Communities Research Funding Area Grant Ready grant awarded to Kerryn E. Pike.

## Conflicts of Interest

The authors declare no conflicts of interest.

## Supporting Information

Additional supporting information can be found online in the Supporting Information section.

## Supporting information


**Supporting Information** Supporting Information A: Memory Self‐Efficacy Questionnaire. Supporting Information B: Follow‐up Evaluation Form (adapted from [11] to only include items relevant to this study).

## Data Availability

The datasets generated and analysed during the current study are not publicly available due to ethics requirements but are available on reasonable request.
