# Supplementary material for: A Mixed‐Methods Investigation of the Role of Sense of Community on Older Adults’ Memory Self‐Efficacy and Control Beliefs Following an Online Group Memory Intervention
Source: J Aging Res. 2026 Jul 23;2026:1022137. doi: 10.1155/jare/1022137 (PMC13392815; doi:10.1155/jare/1022137)

**Supplementary Material**

**Supplementary Material A**

Memory Self-Efficacy Questionnaire


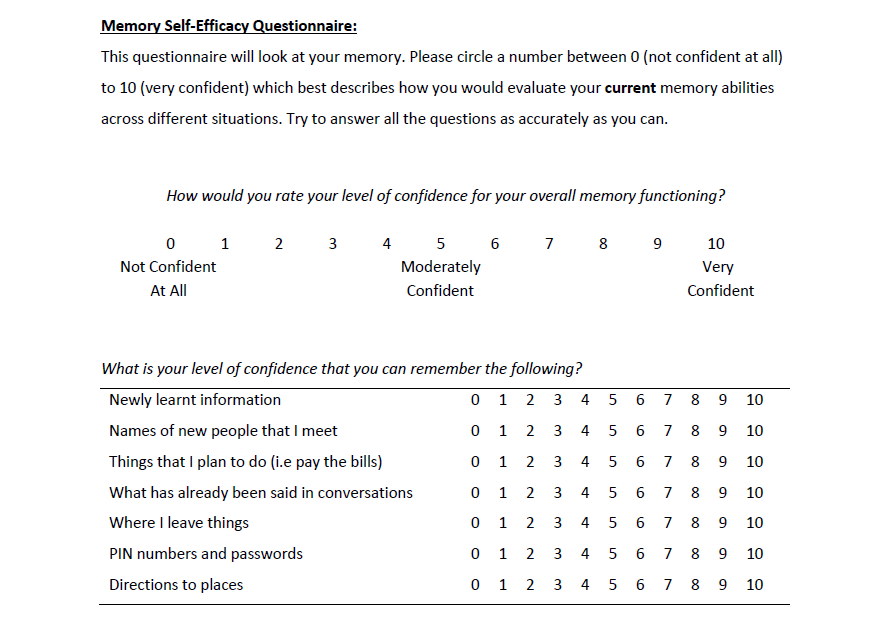


**Supplementary Material B**

Follow Up Evaluation Form (adapted from [11] to only include items relevant to this study)
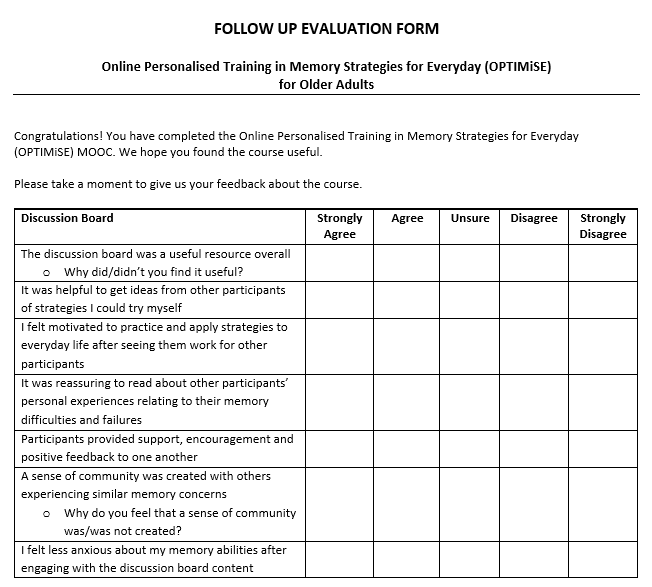

Supplement: Supplementary file 1 — Supporting Information Supporting Information A: Memory Self‐Efficacy Questionnaire. Supporting Information B: Follow‐up Evaluation Form (adapted from [11] to only include items relevant to this study). [file JARE-2026-1022137-s001.docx]
